# Bosentan, a drug used in the treatment of pulmonary hypertension, can prevent development of osteoporosis

**DOI:** 10.22038/ijbms.2021.54152.12172

**Published:** 2021-07

**Authors:** Duygu Köse, Ahmet Köse, Zekai Halıcı, Elif Çadırcı, Taha Tavacı, Muhammed Ali Gürbüz, Adem Maman

**Affiliations:** 1 Clinical Research, Development and Design Application, and Research Center, Ataturk University, Erzurum, Turkey, 905074704150; 2 University of Health Sciences, Faculty of Medicine, Department of Orthopedics And Traumatology, Erzurum, Turkey, 905066330520; 3 Clinical Research, Development and Design Application and Research Center, Ataturk University, Erzurum, Turkey, 905323868884; 4 Faculty of Medicine, Department of Pharmacology, Atatürk University, Erzurum, Turkey, 905362328001; 5 Faculty of Medicine, Department of Pharmacology, Atatürk University, Erzurum, Turkey, 9005059177816; 6 Faculty of Medicine, Department of Histology And Embryology Department, Atatürk University, Erzurum, Turkey, 905522265686; 7 Faculty of Medicine, Department of Nuclear Medicine, Atatürk University, Erzurum, Turkey, 905063661925

**Keywords:** Bone, Bosentan, Endothelin, Endothelin A receptor, Osteoporosis

## Abstract

**Objective(s):**

We examined the antiosteoporotic effect of bosentan (Bose) by radiographic, histopathological, and molecular methods.

**Materials and Methods:**

Rats were divided into 4 groups of 8 rats each: one control (Sham), one osteoporosis only (OP), and two osteoporosis groups treated with Bose doses of 50 and 100 mg/kg (OP+Bose50, OP+Bose100). Six weeks later, Bose was administered for eight weeks to animals undergoing ovariectomy. The left femoral bone of the rats was evaluated in vitro after surgical removal. Bone mineral density (BMD) was analyzed by Dual-energy X-ray absorptiometry (DEXA). Endothelin 1 (ET-1), ET-A, and ET-B expressions were examined by real-time polymerase chain reaction (real time-PCR). In addition, bone tissue was evaluated histopathologically.

**Results:**

Compared with the osteoporosıs group, Bose significantly increased BMD values at both 50 and 100 mg/kg doses. ET-1 mRNA levels were significantly higher in the OP group than in the Sham group, while ET-1 mRNA levels were significantly lower in Bose treatment groups. ET-A mRNA levels were significantly lower in the OP group than in the Sham group, while ET-A mRNA levels were significantly higher in Bose treatment groups. Histopathological results supported the molecular results.

**Conclusion:**

Our study is the first to demonstrate the molecular, radiological, and histopathological effects of Bose in preventing osteoporosis in rats.

## Introduction

Osteoporosis is a preventable systemic disease that is very common all over the world and is characterized by deterioration of bone microstructure and decreased bone mass (1). Important reasons that increase and accelerate the development of osteoporosis are estrogen deficiency, inflammatory diseases, and aging (2). Experimental animal models are very good for osteoporosis prevention and treatment studies. One of the most common and first-choice osteoporosis models used for such studies is the ovariectomized rat model (3). The ovariectomized rat model of osteoporosis produces bone loss due to estrogen deficiency and shows clinical signs of postmenopausal osteoporosis (4). Significant bone loss is observed in the humerus, femur, tibia, and spine (5). In rats the proximal femur shares many histoanatomical similarities with humans (6). 

After ovariectomy, bone resorption in rats begins to surpass bone formation and bone loss begins to occur, and significant early bone loss in the trabeculae in the femoral neck occurs approximately 30 days after ovariectomy (7-9). Clinically, bone mineral density (BMD) measurement is used to assess osteoporosis. BMD is measured by dual-energy X-ray absorptiometry (DEXA). Osteoporosis diagnosis in clinical settings and the gold standard method used to evaluate treatment efficacy is DEXA (10). In osteoporosis, while bone mineral BMD decreases in measurements performed by DEXA, micro-examination of the bone reveals decreased bone mass and trabecula/spicule thickness, and increased alveolar volume (11).

Endothelin 1 (ET-1) is one of the strongest vasoconstrictor substances (12). ET-1 is synthesized from various cells such as smooth muscle cells, macrophages, and bone cells (13-18). It is known that there is an important relationship between ET-1 and BMD (19). An increase in ET-1 can cause an increase in osteopenia (20). ET-1 is known to increase osteoclast-induced bone resorption and on the other hand, it has been shown to stimulate osteoblast proliferation and differentiation in *in vitro* studies (21-23). ET-1 has four receptors (ET-A, ET-B1, ET-B2, and ET-C) (17, 18). Bosentan is an approved drug for the treatment of pulmonary artery hypertension and is a non-selective endothelin receptor antagonist for ET-A and ET-B receptors.

We investigated for the first time, the antiosteoporotic effect of bosentan with radiographic, histopathologic, and molecular methods. 

## Materials and Methods

***Animals***

Thirty-two female albino Sprague-Dawley rats (10-12 weeks old) weighing between 240 and 260 g were taken from Atatürk University (ATADEM) Medical Experimental Research Center and randomly divided into 4 groups. At the end of the experiment (14 weeks), 5 animals from each group were evaluated except the animals that died or were injured for various reasons. The experiments were carried out under normal temperature conditions (22 ^°^C) and controlled light conditions (12 hr light/dark cycle), and the environment was regularly ventilated. The use and care of laboratory animals were approved by the Atatürk University Institutional Animal Care and Use Committee and the experiments were carried out according to international guidelines (Date, 27.06.2019; Meeting number, 7; Decision number, 114; Document number, 42190979-000-E.1900179333). Rats were housed in plastic cages with a sawdust bottom, and standard rat food and tap water were provided *ad libitum*.

***Chemicals***

Bosentan was purchased from Actelion Pharmaceuticals (Allschwil, Switzerland), ketamine (Ketalar 500 mg/10 ml) was purchased from Pfizer, Turkey, xylazine (Basilaz 2%) was purchased from Biotek, Turkey, and metamizole sodium (Novalgin 500 mg/ml injectable preparations ) was purchased from Sanofi-Aventis, Turkey. All other chemicals for laboratory experiments were purchased from Sigma and Merck, Germany.

***Ovariectomy surgery***

Bilateral ovariectomy was performed on 24 animals (24, 25). Animals were anesthetized intraperitoneally with 80 mg/kg ketamine+8 mg/kg xylazine. After a longitudinal incision (0.5-1 cm) was made in the middle of the lower abdomen, the ovaries were removed with a small peritoneal incision.

***Drug applications***

The rats were divided into 4 groups, including 8 rats in each group: control, osteoporosis, and 2 osteoporosis groups treated with bosentan.

- Sham: Surgical control group
- Osteoporosıs (OP): Ovariectomized group
- OP+Bose50: Bosentan administered 50 mg/kg
- OP+Bose100: Bosentan administered 100 mg/kg

Drug administration was started six weeks after ovariectomy. Bosentan was dissolved in 1 ml of distilled water and administered orally once a day for eight weeks. Only 1 ml of distilled water was given to the Sham and Osteoporosıs groups.

***Dual-energy X-ray absorptiometry (DEXA) estimations***

The left femoral bone of rats was assessed *in vitro* after surgical removal. BMD was analyzed by DEXA using Discovery Wi (Hologic Inc. Bedford, MA, USA). The same investigator performed each measurement and all analysis was performed using the same GYregion (ROI) window size.

***Histopathological analysis***

*Routine Histological and Hematoxylin Eosin (HE)Procedure:*

Hard bone tissue samples in fixative were thrown into 10% nitric acid and tissue softening was followed with a needle. The tissues were followed up after 2-10 days of decalcification. The tissues obtained after the follow-up were blocked with a paraffin solution at 67 ^°^C in a Leica EG1160 blocking device. The blocked tissues were cooled at -4 ^°^C. In a Leica Autostainer XL automatic staining and sealing device, HE staining was performed by an automated procedure. The final stage in the automatic painting and sealing device is closing by dropping the lid and the preparations are made ready for examination.

After the necessary studies, histopathological studies were evaluated as follows. In a light microscope, the following analyzes were performed to evaluate HE stained preparations during the examination stage (25, 26).

- old bone mass
- trabecula/spicule thickness
- alveolar volume
- new bone formation

Five fields for each femur were evaluated at 40x magnification. In grading the severity of the changes; grade 0: - (0% negative), grade 1: + (0–33% positive), grade 2: ++ (33–66% moderately positive), and grade 3: +++ (66–100% severe) positive ) were evaluated (27, 28)(Table 1).

***Molecular studies***

Bone ET-1, ET-A, ET-B, and mRNA expression levels were evaluated by real-time polymerase chain reaction (Real Time-PCR). For this, respectively, homogenization of bone tissues, RNA isolation, cDNA synthesis, and quantitative determination of mRNA expressions were made.

***Real Time-PCR***

*Ribonucleic acid (RNA) extraction from rat femoral tissue*

Tissues (20 mg) were homogenized with nitrogen using a Tissue Lyser II (Qiagen). RNA extraction was performed on a QIAcube. The total amount of mRNA was measured at 260 nm using a nano-drop spectrophotometer (EPOCH Take 3 Plate, Biotek). The obtained RNA was stored at -80 ^°^C under the required conditions.

*Reverse transcriptase reaction and complementary DNA (cDNA) synthesis*

Production of cDNA from RNA was achieved with the High Capacity cDNA Reverse Transcription Kit. For cDNA synthesis, 10 μl RNA was used in each reaction with a Thermal Cycler (Applied Biosystem) according to temperature values. The amount of cDNA was determined by a nanodrop spectrophotometer (EPOCH Take3 Plate, Biotek) and the obtained cDNA was stored at -20 ^°^C. For the cDNA synthesis reaction, 10 µl of total RNA, 2 µl of 10 X RT Buffer, 0.8 µl of a mixture of 25 X dNTPs, 2 µl of 10 X RT Random Primers, 1 Multil MultiScribe Reverse Transcriptase, and 4.2 µl of diethylpyrocarbonate H_2_O were used. 

*Quantitative determination of ET-1, ET-A, and ET-B mRNA expressions by Real-Time PCR*

We investigated ET-1, ET-A, and ET-B expressions to understand the healing process by comparing groups with or without bosentan treatment. ET-1, ET-A, and ET-B mRNA expression were achieved using Taq Man Gene Expression Master Mix. b actin was used as the reference gene. Amplification and quantification were performed using the StepOne Plus Real-Time PCR System (Applied Biosystems). The following TaqMan® Gene Expression Assays for 200 ng cDNA were continued by pipetting as described below for 40 cycles. X µl (X=9) cDNA (200 ng), 10 µl TaqMan Master Mix, and 1 μl assay were used for pipetting and completed to 20 µl with RNase free H_2_O. CT (cycle threshold) is the number of cycles (threshold cycle) in which the amount of fluorescent signal in real-time PCR experiments exceeds the minimum value (threshold value) required to be observed. Ct values ​​were automatically converted into ∆∆Ct and the results were statistically evaluated in SPSS 25.00 software program (29). 

***Statistical methods***

Data are expressed as mean standard deviation (SD). A one-way analysis of variance (ANOVA) and Duncan tests were performed to test any differences between groups. *P*<0.05 was considered statistically significant.

**Table 1:** H&E staining scores of femur bone tissues of Sham, Osteoporosis, and Bose groups

Groups	Old bone mass	Trabecula/ spicule thickness	Alveolar volume	New bone formation
Sham	+++	+++	+	+
Osteoporosis	+	+	+++	+
Op+bose50	++	++	+	++
Op+bose100	++	+++	+	+++

Grade 0: - (0% negative), Grade 1: +(0–33% mild positive), Grade 2: ++ (33–66% moderate positive), and Grade 3: +++(66–100% severe positive)

HE: Hematoxylin Eosin Staining, OP: Osteoporosis, Bose: Bosentan

**Figure 1:**
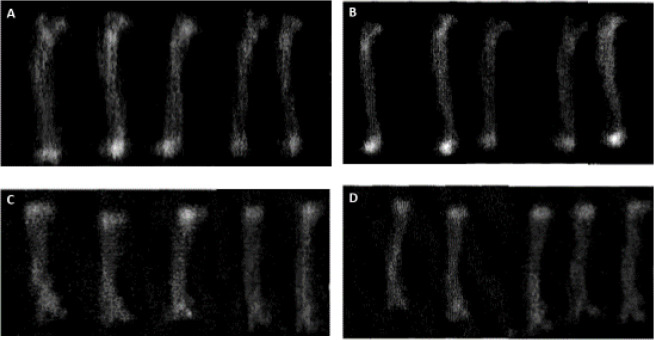
Left femoral bone DEXA images of Sham (1A), Osteoporosis (1B), and Bose groups (1C, D)

Bose: bosentan, OP: osteoporosis, DEXA: DEXA Dual-energy X-ray absorptiometry

**Figure 2:**
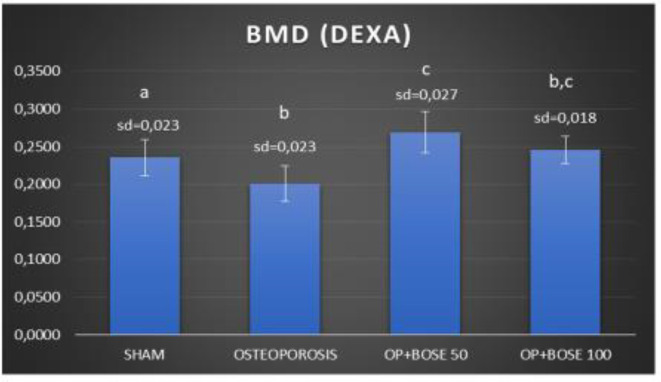
Left femoral bone BMD measured with DEXA results of Sham, Osteoporosis, and Bose groups. Means which have the same letter are not significantly different from the Duncan test (*P=*0.05) Results are expressed as mean±SD. Means which have the same letter are not significantly different from the Duncan test (*P=*0.05)

Bose: bosentan, OP: osteoporosis, DEXA= DEXA Dual-energy X-ray absorptiometry, BMD= bone mineral density

**Figure 3:**
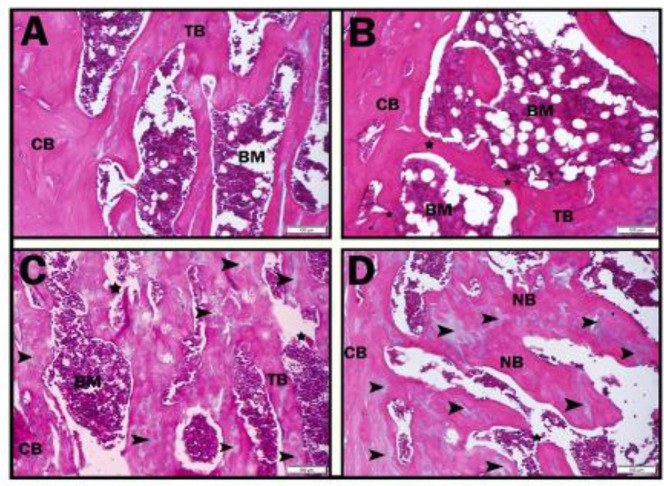
H&E staining findings of femur bone tissues of Sham, Osteoporosis, and Bose groups. Figure a; Sham, Figure b; Osteoporosis, Figure c; OP+Bose50, Figure d; OP+Bose100

**Figure 4:**
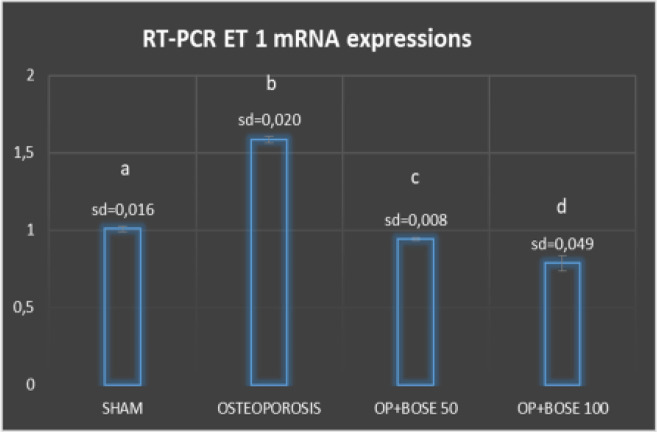
Relative mRNA expression levels of ET-1 of femur bone tissue of Sham, Osteoporosis, and Bose groups. Expression of ET-1 was detected by quantitative RT-PCR analysis. The relative expression levels were calculated by the 2(−ΔΔCT) method. β-Actin was used as the reference gene. Results are expressed as mean±SD. Means which have the same letter are not significantly different from the Duncan test (*P=*0.05)

ET= endothelin, Bose: bosentan, OP: osteoporosis, SD= standart deviation

**Figure 5:**
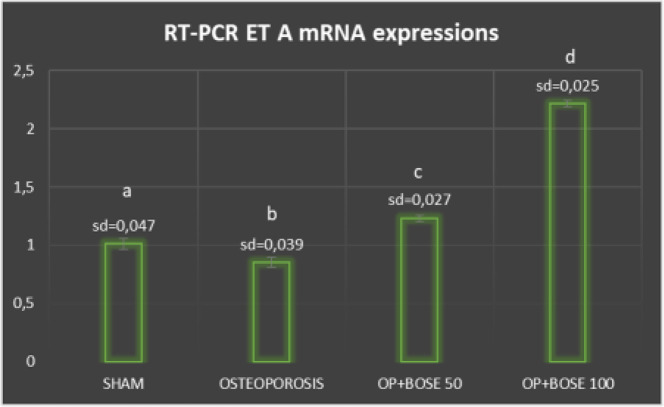
Relative mRNA expression levels of ET-A of femur bone tissue of Sham, Osteoporosis, and Bose groups. Expression of ET-1 was detected by quantitative RT-PCR analysis. The relative expression levels were calculated by the 2(−ΔΔCT) method. β-Actin was used as the reference gene. Bose: Bosentan, OP: osteoporosis. The means which have the same letter are not significantly different from the Duncan test (*P=*0.05). Results are expressed as mean±SD

ET= endothelin, Bose: bosentan, OP: osteoporosis, SD= standart deviation

## Results

***Bone mineral density***

Eight weeks after ovariectomy, there was a significant decrease in femoral BMD values (g/cm2) in the Osteoporosıs group compared with the Sham group (Figures 1A, B, and 2). When compared with the Osteoporosıs group, Bose significantly increased BMD values both in 50 and 100 mg/kg doses (*P*<0.05) (Figures 1B, C, D, and 2).

***Histopathology results***

*Hematoxylin and eosin staining results*

The histopathological examinations performed by HE staining in the femur tissues are described below:

Sham: In the overall appearance of Trabecular (TB), cortical bone (CB) integrity, and bone marrow (BM) no anomaly was detected . The volume of the cancellous bone spicules, the dimensions of the bone marrow areas, and the density/staining characteristics of the bone matrix were normal (Figure 3A).

Osteoporosis: Osteoporotic findings are evident: decrease in both cortical and cancellous bone mass due to osteoporosis and increase in alveolar spaces containing bone marrow was observed. In some regions, a decrease in the thickness of the spongiosis spicules and ruptures in the continuity of the spicule network (stars) were observed (Figure 3B).

OP+Bose50: Numerous indentations-protrusions due to possible osteoclastic activity were observed on the surfaces of bone spicules facing the alveolar spaces. Damage to the joints associated with the trabecular bone continues. When the eosinophilic staining properties of the bone line were examined, new bone formation areas had fragmented and small surface areas. It was found that osteoid deposition was at the initial level. Bluish-stained regions where chondroblasts are located show callus tissues in which ossification has not begun. (Figure 3C).

OP+Bose100: When compared with the OP+Bose50 group, it was analyzed that trabecular bone damage decreased considerably and the thickness of bone spicules increased due to new bone formation. It was observed that the high-dose active ingredient did not cause a significant increase in new bone development and osteoid accumulation, although it decreased the level of osteopenic damage (Figure 3D).

***Real-time PCR results***

*ET-1, ET-A, and ET-B* *mRNA levels*

Using RT-PCR, we investigated ET-1, ET-A, and ET-B mRNA expressions in the rats’ femur bone tissue. ET-1 mRNA levels were significantly higher in the Osteoporosis group when compared with the Sham group (*P*<0.05) (Figure 4). In contrast, ET-1 mRNA levels were significantly lower in the Bose treatment groups (*P*<0.05). ET-A mRNA levels were significantly lower in the Osteoporosis group when compared with the Sham group (*P*<0.05) (Figure 5). In contrast, ET-A mRNA levels were higher significantly in the Bose treatment groups (*P*<0.05) (Figure 5). No significant difference in statistical terms could be found when ET-B mRNA expressions of the treatment groups were compared with those of the Sham or Osteoporosis groups (*P*>0.05).

## Discussion

In our study, after 6 weeks from ovariectomy operation when significant osteoporotic changes started to occur, we started bosentan application in treatment groups. We applied bosentan to the treatment groups for 8 weeks. At the end of 14 weeks, while BMD decreased significantly in the ovariectomized group, it significantly increased in the bosentan treatment groups.

Trabecular and cortical bones are very close to the vascular endothelial cells. Endothelial cell products like ET-1 can affect bone cell function. Studies have shown that ET-1 stimulates the proliferation of osteoblastic cells and the effect of ET-1 is dependent on its binding to the ET-A receptors (30). Although normal levels of ET-1 are potent regulators of human bone cell metabolism, in many studies in the literature, it is shown that patients with osteoporosis have increased serum ET-1 levels (31, 32). Our study supported these studies. Increased expression of bone ET-1 in the Osteoporosis group indicates that ET-1 plays a role in the pathophysiology of osteoporosis in bone tissue. The effect of bone ET-1 on osteoporosis was limited with bosentan. Non-selective endothelin receptor antagonist for ET-A and ET-B receptors and accordingly, the pathological bone ET-1 levels increased with osteoporosis returned to normal levels with the reduction of bone ET-1 in both doses of bosentan. It is known that bone ET-1 expressions increase pathologically in bone fractures and ET-1 antagonist bosentan treatment reduces the increasing pathological bone ET-1 expressions to normal and increases fracture healing (33). Similarly, in our study, there was a significant increase in BMD scores in both of the bosentan treatment groups compared with the Osteoporosis group. While down-regulation of bone ET-A receptors was observed due to the increase of bone ET-1 in the Osteoporosis group, up-regulation was observed in bone ET-A receptors due to ET-1 antagonism in bosentan treatment groups. 

In histopathologic analysis, decreased bone mass and trabecula/spicule thickness and increased alveolar volume with osteoporosis improved with bosentan treatment. Old bone mass and new bone formation increased significantly in the bosentan treatment groups compared with the Osteoporosis group. It is known that bosentan increases new bone formation in fracture healing (33). Similarly, it increased the formation of new bone in osteoporosis, as well as preserving the old bone mass.

Bosentan significantly prevents the occurrence of osteoporosis. Osteoporosis, which is a very difficult disease to treat, is also a preventable disease and is easier to prevent than to cured. Since bosentan is a drug used in the treatment of pulmonary hypertension and patients who are treated for pulmonary hypertension may be bedridden, choosing bosentan treatment in these patients during this period will significantly reduce the development of osteoporosis.

## Conclusion

Our study is the first to demonstrate molecular, radiological, and histopathological effects of bosentan in preventing osteoporosis in rats. In the treatment of pulmonary hypertension, bosentan may be a good treatment choice in patients at the risk of developing osteoporosis.
